# Hydroxyapatite/L-Lysine Composite Coating as Glassy Carbon Electrode Modifier for the Analysis and Detection of Nile Blue A

**DOI:** 10.3390/ma15124262

**Published:** 2022-06-16

**Authors:** Jimmy Julio Kouanang Ngouoko, Kevin Yemele Tajeu, Ranil Clément Tonleu Temgoua, Giscard Doungmo, Ingo Doench, Arnaud Kamdem Tamo, Théophile Kamgaing, Anayancy Osorio-Madrazo, Ignas Kenfack Tonle

**Affiliations:** 1 Department of Chemistry, Faculty of Science, University of Dschang, Dschang P.O. Box 67, Cameroon; ngouoalex@yahoo.com (J.J.K.N.); tasergekev@yahoo.fr (K.Y.T.); raniltemgoua@yahoo.fr (R.C.T.T.); theokamgaing@yahoo.fr (T.K.); 2 Higher Teacher Training College, University of Yaoundé 1, Yaoundé P.O. Box 47, Cameroon; 3 Institute of Inorganic Chemistry, Christian-Albrechts-Universität zu Kiel, Max-Eyth-Straβe 2, 24118 Kiel, Germany; gdoungmo@ac.uni-kiel.de; 4 Laboratory for Bioinspired Materials BMBT, Institute of Microsystems Engineering IMTEK-Sensors, University of Freiburg, 79110 Freiburg, Germany; ingo.doench@imtek.uni-freiburg.de; 5 Freiburg Center for Interactive Materials and Bioinspired Technologies FIT, University of Freiburg, 79110 Freiburg, Germany; 6 Freiburg Materials Research Center FMF, University of Freiburg, 79104 Freiburg, Germany

**Keywords:** hydroxyapatite, lysine, inorganic–organic composite, glassy carbon electrode, electrode coating, electrochemical analysis, Nile blue A

## Abstract

An amperometric sensor was developed by depositing a film coating of hydroxyapatite (HA)/L-lysine (Lys) composite material on a glassy carbon electrode (GCE). It was applied for the detection of Nile blue A (NBA). Hydroxyapatite was obtained from snail shells and its structural properties before and after its combination with Lys were characterized using X-ray diffraction (XRD), Fourier-transform infrared (FTIR) spectroscopy, scanning electron microscopy (SEM), and Brunauer–Emmett–Teller (BET) surface area analyses. The coupling of Lys to HA was attributed to favorable interaction between negatively charged -COO^−^ groups of Lys and divalent ions Ca^2+^ of HA. Electrochemical investigations pointed out the improvement in sensitivity of the GCE/Lys/HA sensor towards the detection of NBA in solution. The dependence of the peak current and potential on the pH, scan rate, and NBA concentration was also investigated. Under optimal conditions, the GCE/Lys/HA sensor showed a good reproducibility, selectivity, and a NBA low detection limit of 5.07 × 10^−8^ mol L^−1^. The developed HA/Lys-modified electrode was successfully applied for the detection of NBA in various water samples.

## 1. Introduction

Nile blue A (bis [5-Amino-9-(diethylamino)benzo[a]phenoxazin-7-ium] sulphate) is an azo dye of the phenoxazine family [1]. It finds applications in histology and medicine for the detection of micro-organisms [2], as well as in photodynamic therapy for the treatment of malignant tumors [3,4]. It is also applied in dye-sensitized solar cells [5]. However, it is a mutagen [6] and carcinogen [7], and for these reasons, prohibited as a food additive. The presence of this dye in water is harmful to microbial life [8]. Due to the risks presented by this dye, its elimination in water and in various matrices is an active field of research. For that purpose, various techniques have been used, including adsorption [9], chemical degradation [10], and absorption [11,12]. Furthermore, the quantification of NBA at trace level is relevant in analytical and environmental sciences. In these fields, solid electrodes chemically modified by convenient inorganic materials, likely to display great affinity for a target species, are usually used. Some common examples of such materials are silica [13,14], clay minerals [15,16], various carbon derivatives [17,18,19,20], and metal oxides [21,22]. Furthermore, additional studies also have highlighted the use of conjugated organic polymers (conductive polymers) as electrode modifiers in the fabrication of modified electrodes for the electroanalysis of various analytes [23,24]. Conducting polymers are organic compounds with considerable flexibility and an extended π-orbital system, through which electrons can move from one end of the polymer to the other. They are among the most relevant and widely used materials for sensor modification due to their unique physical and chemical properties, such as tunable architecture and versatility, good stability, and sensitivity [25,26,27,28]. The resort to these materials aims at improving either the sensitivity or the selectivity of the bare solid electrode. In recent years, the scientific community in analytical electrochemistry has shown great interest in the development of hydroxyapatite-based sensors [29,30,31]. Hydroxyapatite is a low-cost material that can be produced from high calcium phosphate biominerals present, for example, in seashells and animal bones [32,33]. It is a phosphate mineral with the formula Ca_5_(PO_4_)_3_(OH), usually written Ca_10_(PO_4_)_6_(OH)_2_ to underline the fact that a dimer is present in one unit cell [34]. The acid-base properties, ion-exchange capability, and adsorption ability capacity of hydroxyapatite (HA) have boosted the development of electrochemical sensors wherein HA serves as adequate electrode material. Thus, El-Mhammedi et al. [35] used it to modify a carbon paste electrode, which was then applied for the detection of para-nitrophenol. Yin et al. [36] also detected 4-nitrophenol, using a glassy carbon electrode modified with HA nanopowder. Kanchana and Sekar [37] reported the exploitation of the same material as the GCE modifier for the determination of folic acid. Although these works have been relevant, the poor electron transfer capacity associated with insufficient selectivity are known as the drawbacks of sensors based on pure HA. This has further prompted the search for additional compounds to be combined with HA to yield more efficient sensing devices. Along these lines, Kanchana and Sekar [38] proposed an electrochemical sensor of uric acid, based on EDTA/HA nanoparticles. The electroanalysis of both diquat and lead ions was successfully achieved by Tchoffo and coworkers [31,39], using a glassy carbon electrode modified with a hybrid material from HA and β-cyclodextrin.

**Scheme 1:**
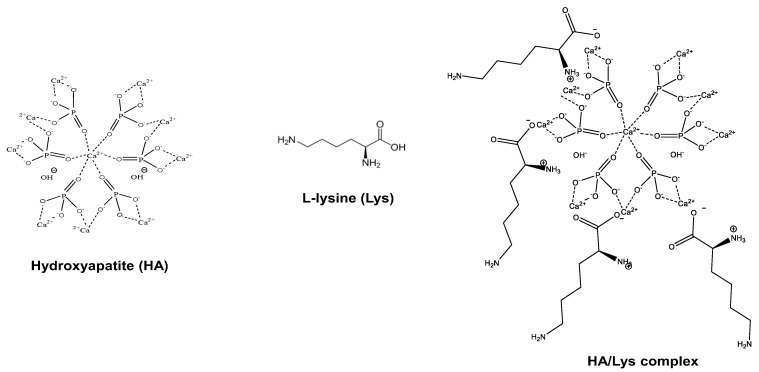
Chemical structures of hydroxyapatite (HA) and L−lysine (Lys), as well as a simplified schematic representation of the hydroxyapatite/L−lysine (HA/Lys) complex.

The present work focused on the synthesis of HA powder from snail shells and the further preparation of HA/L-lysine composite material useful as electrode coating for the detection of Nile blue A (NBA) in solution by electrochemical analysis. L-lysine bears amine groups, which are expected to display, upon protonation, strong affinity with NBA. After its preparation, the Lys/HA composite material was characterized by several physicochemistry techniques. Then, the HA/Lys composite was deposited as a thin film on the active surface of a glassy carbon electrode (GCE) for the voltammetric analysis of NBA by means of cyclic voltammetry (CV), followed by the detection of the same analyte by differential pulse voltammetry (DPV). Key parameters affecting the amperometric response of the sensor were investigated to obtain the best NBA analysis conditions, which were successfully applied for the quantification of NBA in a spring water sample. Scheme 1 below highlights the chemical structure of hydroxyapatite (HA) and that of L-lysine (Lys), as well as a simplified schematic representation of the structure of the Lys/HA complex, including the different functional groups available for interactions with the target analyte (Nile blue A).

## 2. Materials and Methods

### 2.1. Reagents and Chemicals

All chemicals were used without further purification. Na_2_HPO_4_ (98%) and KH_2_PO_4_ (99%) were obtained from BDH. NaOH, EDTA, Nile blue A (98%), L-lysine, and methyl orange were purchased from International Fisher Scientific. Caffeine (99%), toluidine blue, Ni(NO_3_)_2_.6H_2_O (98%), Cd(NO_3_)_2_.4H_2_O (98%), and ascorbic acid were obtained from Sigma-Aldrich. Pb(NO_3_)_2_ (99%) was purchased from VWR Chemicals BDH and Cu(HCO_2_)_2_.2H_2_O (99%) from Merck Chemicals GmbH. Citric acid monohydrate and HCl (36%) were purchased from J.T. Baker and Pronalys AR, respectively. The phosphate buffer solutions used in this work consisted of a mixture of monobasic dihydrogen phosphate and dibasic monohydrogen phosphate. By varying the amount of each salt, we prepared a range of phosphate buffers with pHs between 5.0 and 9.0. Analytical solutions of NBA at various concentrations were obtained by dilution from a standard solution of a concentration of 0.01 M, using doubly distilled water.

Specimens of snail shells were collected from a local market in downtown Nkongsamba (Cameroon). The raw snail shells were washed with water, rinsed with distilled water, and dried at room temperature for two weeks. They were exploited to yield hydroxyapatite, as described in the next section.

### 2.2. Preparation of Hydroxyapatite Powder

The preparation was performed according to a method published by Shavandi et al. [40], with slight modifications. Thus, the calcination of the snail shells was achieved at 1000 °C for 90 min, in an electrical furnace at a heating rate of 5 °C min^−1^. The calcinated product was then crushed using a mortar, then sieved to obtain a white powder (with a particle diameter less than 25 µm). To 2.8 g of calcinated shell, 50 mL of 0.1 M EDTA was added to yield a solution of 0.1 M Ca-EDTA complex. To that solution and under stirring, 50 mL of 0.06 M Na_2_HPO_4_ was added (4 mL min^−1^). The obtained mixture was stirred for 120 min, maintained at a pH around 13. Upon drying in an oven for 12 h, a milky white powder was obtained.

### 2.3. Preparation of Hydroxyapatite/L-Lysine (HA/Lys) Modified Working Electrode

Before modification, the surface of the glassy carbon electrode (GCE) (3 mm in Ø) was polished with alumina slurries of different sizes (1, then 0.5 μm) on billiard cloth and placed in a sonicator for 5 min to eliminate remaining alumina particles. The thin hydroxyapatite (HA) film working electrode was prepared by the drop-coating of 5 µL of a dispersion obtained by sonication for 30 min, and of 5 mg L-lysine with various amounts of HA (0, 1, 2, 3, and 4 mg), in 1 mL of double distilled water. The modified electrodes were dried in an oven at 80 °C for 5 min. In this manuscript, they are denoted: GCE/HA, GCE/Lys, and GCE/Lys/HA, for the GCEs modified with HA, Lys, and Lys/HA composite, respectively.

### 2.4. Material Characterization

The synthesized hydroxyapatite (HA), L-lysine (Lys), and the Lys/HA composite materials were characterized by various physicochemical techniques.

#### 2.4.1. X-ray Diffraction (XRD)

X-ray diffraction patterns were collected using a Stoe Stadi-P X-ray powder diffractometer, with Cu Kα1 radiation (*λ* = 1.54056 Å, gemonochromator, flat sample). The data were collected in the 2*θ* angle ranging from 5° to 70°, with a scanning speed of 1.5° min^−1^.

#### 2.4.2. Fourier-Transform Infrared (FTIR) Spectroscopy

FTIR spectra were registered on a genesis FTIRM spectrometer (ATI Mattson), equipped with a DTGS (deuterated triglycine sulfate).

#### 2.4.3. Brunauer–Emmett–Teller (BET) Analysis

Nitrogen adsorption–desorption isotherms were collected for selected samples using Thermo Electron Corporation, Sorptomatic Advanced Data Processing. Before N_2_ adsorption, the samples were degassed at 307.13 K under a vacuum.

#### 2.4.4. Scanning Electron Microscopy (SEM)

The surface morphology of the L-lysine (Lys), hydroxyapatite (HA), and Lys/HA materials was characterized with a scanning electron microscope (FEI Scios FIB-SEM) at an accelerating voltage of 10 kV. For SEM measurements, the samples were deposited on conductive carbon tabs and coated with gold under a vacuum, using a sputter coater.

### 2.5. Electrochemical Measurements

Electrochemical measurements were carried out at room temperature with μ-Autolab potentiostat (Ecochimie, Holland), employing a conventional three-electrode cell compartment containing the film-modified GCE as the working electrode, the Ag/AgCl (3 M KCl) as the reference electrode (Metrohm), and a steel auxiliary electrode.

Cyclic voltammetry was carried out in a 0.1 M phosphate buffer solution (pH 5.5) containing NBA, in the potential range of −0.9 V to +0.1 V. For stripping analysis of NBA, differential pulse voltammetry in anodic mode was performed at closed circuit in the potential scan range from −0.7 V to 0 V, using the following optimized parameters: pulse amplitude: 95 mV; step potential: 7.5 mV; and equilibrium time: 5 s.

## 3. Results and Discussion

### 3.1. Characterization of Hydroxyapatite (HA) and L-Lysine/Hydroxyapatite (Lys/HA) Hybrid Materials

**Figure 1:**
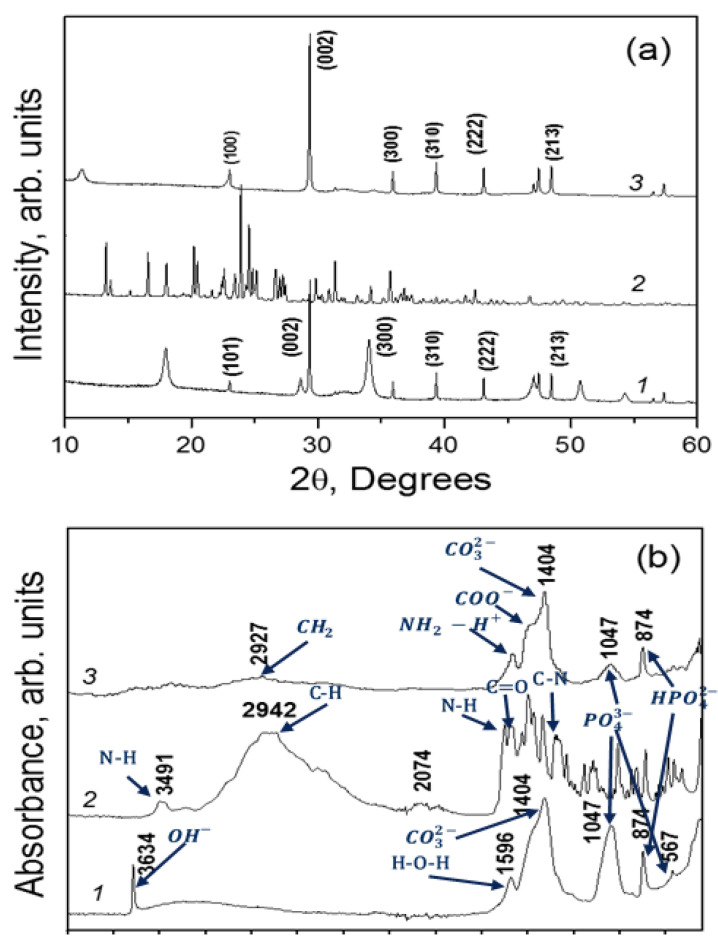
(**a**) Powder X-ray diffraction patterns, and (**b**) FTIR spectra of (1) hydroxyapatite (HA), (2) L-lysine (Lys), and (3) Lys/HA hybrid material.

Figure 1a presents the X-ray diffraction (XRD) patterns of the synthesized HA, pure L-lysine (Lys), and Lys/HA composite materials. On the curve of HA (curve 1), the main diffraction peaks usually observed for pure hydroxyapatite ((101), (002), (300), (310), (222), and (213)) were identified. This was proof that the prepared HA was well-synthesized and constituted a single phase material. However, a broaden background indicates a relatively low crystallinity of the HA. After the addition of lysine to HA, the diffractogram of the Lys/HA hybrid material (curve 3) matched with that of HA, while the intensity of the peaks related to L-lysine were negligible. This could be due to the low amount of the amino acid on the inorganic–organic composite structure. The pattern of pure L-lysine was given in curve 2, for comparison.

This observation led us to estimate the crystallite size and lattice strain [41] of HA before and after its hybridization with L-lysine. Thus, the Williamson–Hall (W-H) equation given by Equation (1) was used [42,43];

(1) $$\beta \cos\theta =\frac{k\lambda }{\tau }+4\varepsilon \sin\theta$$

where *λ* is the wavelength of the CuKα X-ray radiation (*λ* = 1.54178Å), *θ* is the Bragg angle, *ε* is the value of the lattice strain, *τ* is the crystallite size, *β* the full width at half maximum (FWHM) corresponding to (hkl) Bragg’s peak, and *k* is the Scherrer constant usually equal to 0.9.

The W-H plots (*β*cos*θ* vs. 4sin*θ*) for Lys/HA and HA are given in Figure S1 (Supplementary Materials). The crystallite size was determined through the y-intercept, while the lattice strain was derived through the slope of the fitted straight line shown in Figure S1 (Supplementary Materials). The obtained results are given in Table 1.

The observed decrease in lattice strain and increase in crystallite size from HA to Lys/HA were due to an increase in the lattice side after the addition of L-lysine. The degree of crystallization (crystallinity index) was determined based on Equation (2), where *C* is the area of the peaks in the diffraction pattern (crystalline area), and A is the area between the peaks and the background (amorphous area).

(2) $$Crystallinity\;index=\frac{C}{C+A}\times 100$$

The values of *C* and *A* were calculated using Powder X software (version 2017, Germany). From Table 1, crystallinity index values were less than 50%, showing that HA crystallites were practically not affected by the addition of Lys.

Although XRD data did not show the presence of L-lysine on HA, FTIR spectroscopy characterizations were performed to check whether the absorption of amino acid on the surface of HA was effective. The FTIR results are shown in Figure 1b. On the infrared spectrum of synthesized HA (curve 1, Figure 1b), fundamental vibrational modes of the phosphate group were observed: the band at 1047 cm^−1^ was attributed to the phosphate stretching, and the bands at 608, 561, and 476 cm^−1^ were due to the phosphate bending [44,45]. The bands at 3434 and 567 cm^−1^ were attributed to the stretching vibration of OH^−^. The peaks at 1625 and 3334 cm^−1^ were due to absorbed water. The peaks at 1404 and 874 cm^−1^ were attributed to the deformation vibration of CO_3_^2−^ incorporated in the PO_4_^3−^ site and OH^−^ site, respectively; the presence of the peak at 874 cm^−1^ could also have been due to the incorporation of HPO_4_^2−^, characteristic of non-stoichiometric HA [35,36]. These observations confirmed that the synthesized material was pure hydroxyapatite, as revealed by the XRD results. Upon addition of lysine to HA, a comparison between the spectra of pure lysine (curve 2, Figure 1b) and Lys/HA composite (curve 3, Figure 1b) showed bands attributed to the stretching of phosphate (1047 cm^−1^) and the deformation vibration of carbonate ions (1404 and 874 cm^−1^), as well as bands at 1550 cm^−1^ and 1430 cm^−1^ attributed to the symmetric stretching of NH_2_--H^+^ and COO^−^, respectively [46,47]. Furthermore, the band attributed to the stretching of methylene (-CH_2_^−^) was observed (2927 cm^−1^) [37,38]. All these observations suggested the adsorption of lysine on the surface of synthesized HA.

The specific surface area and pore volume of pristine HA, L-lysine, and Lys/HA materials were calculated from the nitrogen adsorption–desorption isotherms, via the Brunauer–Emmett–Teller (BET) and the Barrett–Joyner–Halenda (BJH) methods. The measured specific surface and the pore volume of pristine HA were 46.69 m^2^ g^−1^ and 0.1266 cm^3^ g^−1^, respectively (Table 2). These data decreased to 9.63 m^2^ g^−1^ and 0.0258 cm^3^ g^−1^ when L-lysine was bounded to the surface of the inorganic backbone, meaning that the coupling of L-lysine resulted in the reduction of the porosity of HA. Similar results were already reported by previous works from the literature [48,49], which support the successful adsorption of L-lysine on the surface of HA.

**Figure 2:**
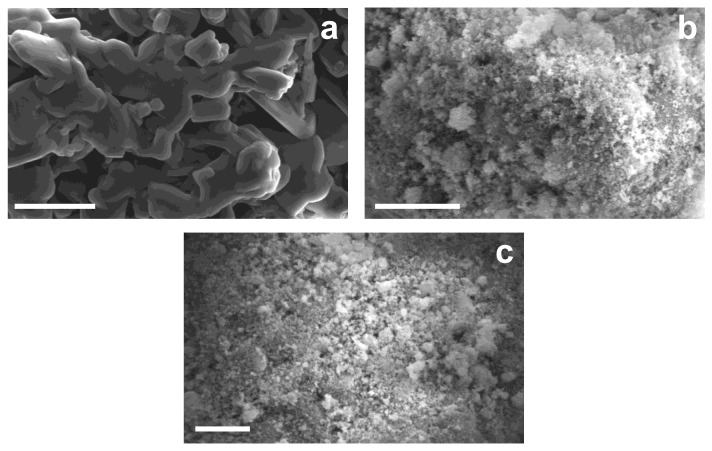
Scanning electron micrographs (SEM) of coating materials: (**a**) L-lysine (Lys); (**b**) hydroxyapatite (HA); and (**c**) hydroxyapatite/L-lysine (HA/Lys) composite. Scale bars: (**a**) 10 µm; (**b**,**c**) 5 µm.

The surface morphologies of the coating materials L-lysine (Lys), hydroxyapatite (HA), and HA/Lys composite, as investigated by scanning electron microscopy (SEM), are shown in Figure 2. The SEM image of the L-lysine material (Figure 2a) showed a smooth surface of low porosity, typical for protein-based materials, with some platelet-like agglomerates. In contrast, the hydroxyapatite surface (Figure 2b) showed micro-sized particles clumped together with interconnected pores. The surface grains were homogeneously small, and the porous sizes were uniform. The addition of lysine to the hydroxyapatite to form the Lys/HA composite (Figure 2c) changed the material morphology compared to that of pure lysine. The surface of the Lys/HA composite (Figure 2c) showed a structure more or less similar to that of the naked hydroxyapatite (as expected for a low lysine content) with a rough surface morphology, a slightly reduced porosity compared to that of HA alone, and foam-like grains with some agglomerated nanocrystals. As for these SEM observations and the above discussed X-ray diffractograms, the HA/Lys might have a relatively low content of organic material, this is, low lysine content. Nevertheless, the addition of lysine slightly decreased the specific surface area and the diameter of the pores (Figure 2), as previously revealed by the absorption and desorption experiments of N_2_ (BET and BJH measurements). Besides, this addition provided new chemical functions and additional active sites, as observed in the FTIR analyses, capable to interact with the target analyte (Nile blue A) in the electrochemical and sorption processes.

### 3.2. Electroanalytical Applications of Lys/HA Composite for Nile Blue A Sensing

#### 3.2.1. Preliminary Study on the Effect of the Working Electrode Modification

**Figure 3:**
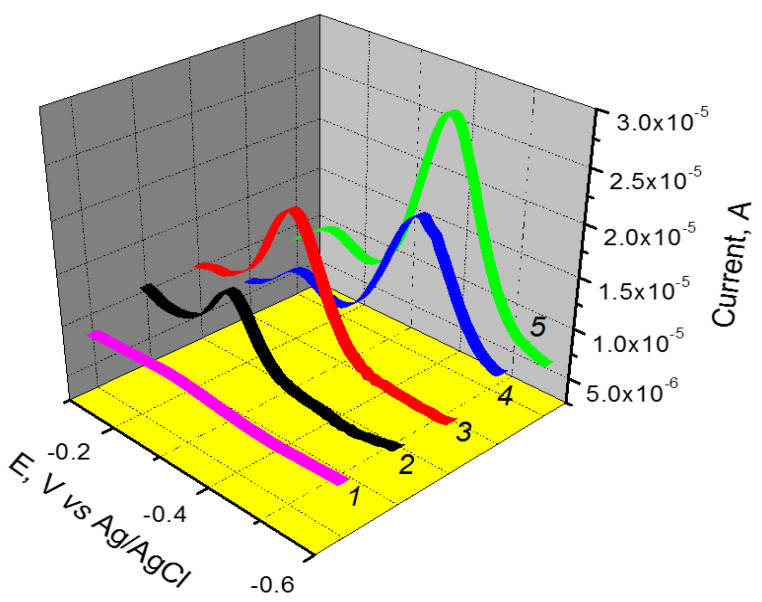
Differential pulse voltammetry (DPV) curves recorded in 0.1 M PBS (pH 5.5) containing 1 µM of NBA on (2) bare GCE, (3) GCE/HA, (4) GCE/Lys, and (5) GCE/Lys/HA. (1) represents the curve recorded in blank electrolyte using GCE/Lys/HA.

Preliminary experiments were performed to establish the possibility of using Lys/HA composite as an electrode modifier for the electroanalysis of NBA. Thus, the performance of the GCE/HA, GCE/Lys, and GCE/Lys/HA towards the detection of NBA was compared. The results obtained are shown in Figure 3. Each electrode gave rise to a well-defined peak in the potential range from −0.6 to −0.1 V. On the bare GCE, a peak current of 6.4 µA was recorded, proving that NBA was electroactive on the GCE. Then, the presence of each modifier improved the performance of the GCE, according to the following ability order: GCE/HA < GCE/Lys < GCE/Lys/HA, with peak currents of 8.8; 12.2, and 13.2 µA, respectively. The presence of HA, on one hand, and Lys, on another hand, on the GCE significantly increased the electrochemical signal of the electrode due to the pre-concentration of NBA dye. By combining HA and Lys on the GCE, the highest current was obtained, meaning that the composite material was more efficient towards the fixation of NBA at the working electrode. To explain these observations, one could reasonably evoke the adsorption of NBA cations on the HA surface and the uptake by lysine through electrostatic attraction between the negative carboxylate ions and protonated NH_2_ groups of NBA. The –NH_3_^+^ groups of lysine can also interact with NH or the aromatic ring of NBA. Explicitly, the Ca^2+^ site of HA can easily bind to negatively charged anionic groups, such as the COO^−^ carboxyl groups carried by lysine. This thus offers the possibility of incorporating biological molecules such as amino acids on the surface of HA. Ozhukil Kollath et al. [50] provided qualitative and quantitative analyses of the L-lysine molecules incorporated on the HA surface and the mechanism of interaction, which demonstrated that the carboxyl group of lysine lends itself well to Coulomb interactions with the Ca^2+^ of hydroxyapatite, which leaves the amino group of lysine available for other reactions, such as interactions with the chemical functions of NBA (for the case of the present work).

In addition, other studies [35,37,51,52,53] also demonstrated that there is a special affinity between HA and amino acids and proteins. This can be explained by the electrostatic attraction forces existing between the carboxylate groups of the amino acids and the Ca^2+^ cations on the surface of HA. Such interactions have also been highlighted in various published works [47,54,55,56,57,58]. Besides, the intermolecular H bonds existing between the N-containing group and the phosphate on the HA surface, as well as the cooperation of several protein functions, Ca^2+^ cations, and phosphate groups on the surface of HA, could explain this affinity. Therefore, amino acids are adsorbed on HA surfaces and maintain their activity due to the good biocompatibility of HA. The good stability and the biocompatibility of HA allow, without ambiguity, to easily incorporate additional chemical functions and physicochemical properties in their structure (in our case, through the incorporation of lysine in the structure of HA), allowing its use as an electrode material for the improvement of the sensitivity and selectivity of resulting electrodes in the electroanalysis of analytes in solution. Moreover, HA is an inexpensive material with high stability, low toxicity, and high abundance. Thanks to these advantages, we fabricated a Lys/HA electrode for the detection of NBA in solution, which improved the electrochemical parameters, such as sensitivity, stability, and selectivity, of the fabricated electrode with respect to the detection electrochemistry of the target analyte (NBA). It is also important to point out the electrocatalytic effect of the modifier, observed through the shift in potential towards negative values compared to the bare GCE electrode. The observations evolved from this section’s conclusion revealed that the GCE modified by a combination of Lys and HA is a prominent tool that can be exploited for the electrochemical analysis and detection of NBA in an aqueous solution. However, it seemed useful to analyze some kinetics aspects taking place at the GCE/Lys/HA, in order to gain insight into the phenomenon occurring in the bulk of this electrode.

#### 3.2.2. Kinetics Studies of GCE/Lys/HA Sensor by Cyclic Voltammetry

**Figure 4:**
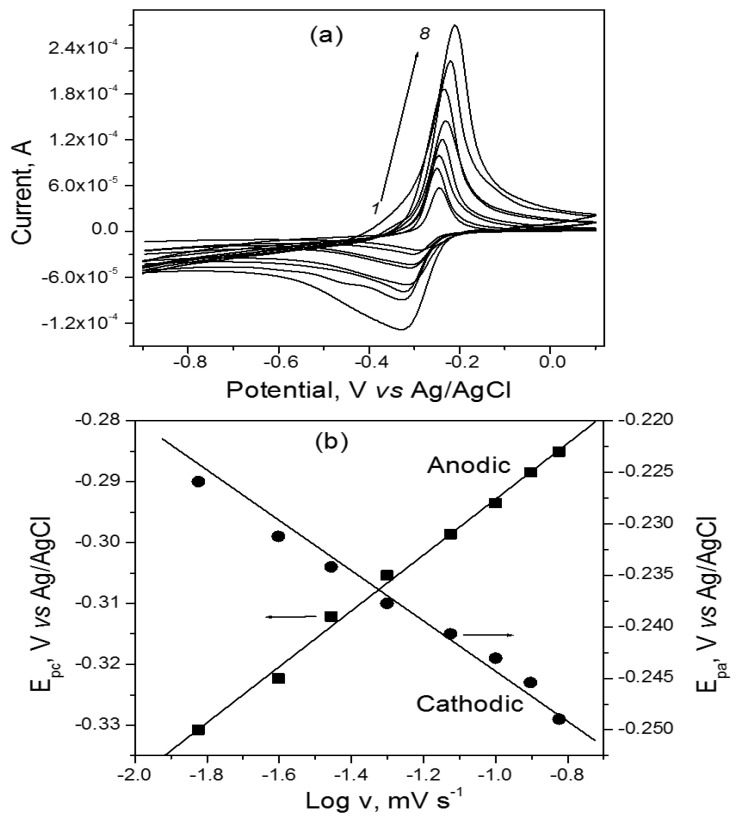
(**a**) Cyclic voltammograms recorded in 0.1 M PBS (at pH 5.5) +1 mM NBA at various scan rates: (1) 15, (2) 25, (3) 35, (4) 50, (5) 75, (6) 100, (7) 125, and (8) 150 mV·s^−1^. The cyclic voltammograms recorded from lowest to highest intensity are those obtained at increasing scan rates between 15 and 150 mV.s^-1^ respectively. (**b**) Plot scan rate potential as a function of the logarithm of scan rate, from the cyclic voltammograms in (**a**).

To determine the heterogeneous electron transfer rate constant and the number of electrons transferred during the oxidation–reduction reaction of NBA at the surface of the GCE/Lys/HA, the effect of scan rate (v) on both the oxidation and reduction peak current of NBA was investigated for v, varied between 15 and 150 mV·s^−1^. The voltammograms obtained in 0.1 M PBS (pH 5.5) containing 1 mM of NBA are shown in Figure 4a.

The electrode exhibited a quasi-reversible system, with peak currents increasing with scan rate. The plot of the peak intensity as a function of scan rate (see Supplementary Materials, Figure S2) was linear, and the regression equations of the straight line obtained in oxidation and reduction directions were, respectively:

Iox(A) = 1.354 × 10^−6^ v(V/s) + 5.163 × 10^−5^ (R^2^ = 0.991) (3)

Ired(A) = −5.969 × 10^−7^ v(V/s) – 1.819 × 10^−5^ (R^2^ = 0.985) (4)

The values of R² close to one showed that both the oxidation and reduction of NBA at the GCE/Lys/HA are adsorption-controlled processes. Such behavior was obtained by Dilek et al. during the electrosynthesis of poly (Nile blue) films on the surface of a glassy carbon disc electrode [59].

The number of electron(s) exchanged and the heterogeneous electron transfer rate constant were determined based on the graph Ep(V) vs. Logv (Figure 4b), and on Laviron Equations (5) and (6) for the quasi-reversible system [60].

(5) $$E_{\mathrm{pa}}=E{}^{\circ}+\left(\frac{2.303\mathrm{RT}}{(1-\alpha )\mathrm{nF}}\right)\mathrm{logv}+\left(\frac{2.303\mathrm{RT}}{(1-\alpha )\mathrm{nF}}\right)\log\left(\frac{\mathrm{nF}(1-\alpha )}{{\mathrm{RTK}}_{s}}\right)$$

(6) $$E_{\mathrm{pc}}=E{}^{\circ}+\left(\frac{2.303\mathrm{RT}}{\alpha \mathrm{nF}}\right)\mathrm{logv}+\left(\frac{2.303\mathrm{RT}}{\alpha \mathrm{nF}}\right)\log\left(\frac{\mathrm{nF}\alpha }{{\mathrm{RTK}}_{s}}\right)$$

where R = 8.314 J.mol^−1^.k^−1^, T = 298 K, and F = 96,487 C.mol^−1^.

The value of the transfer coefficient α was obtained from Equation (7), which represents the ratio of the slope of Equations (5) and (6).

(7) $$\frac{\alpha }{1-\alpha }=\frac{0.0271}{0.03656}=0.741$$

The obtained value of α was 0.426. By reporting this in the slope of either Equation (5) or Equation (6), the number of electrons transferred was found to be n = 1.65, smaller than 2 as in the literature [45]. The heterogeneous electron transfer rate constant Ks was calculated at a scan rate of 50 mV s^−1^ from Equation (8):

(8) $${\mathrm{logK}}_{s}=\alpha \log(1-\alpha )+(1-\alpha )\log\alpha -\log\left(\frac{\mathrm{RT}}{\mathrm{nFv}}\right)-\frac{\alpha (1-\alpha )\mathrm{nF}\Delta E}{2.303\mathrm{RT}}$$

A value of Ks = 0.511 s^−1^ was obtained, indicating that the electron-transfer kinetics are quite fast, despite the process at the electrode being quasi-reversible.

#### 3.2.3. Effect of the Amount of Hydroxyapatite (HA) in the L-Lysine/HA Composite on the Detection of Nile Blue A (NBA)

The amount of hydroxyapatite in the modifier film was expected to affect the response of the electrode. Thus, the variation of the mass of HA in the Lys/HA composite was evaluated and the results are presented in Figure S3 (Supplementary Materials). It was observed that the peak current of NBA increased when the mass of HA in the film was increased between 1 and 3 mg; then it decreased. The observed increase in the peak current with an HA mass between 1 and 3 mg was associated with the presence of more absorption sites in the bulk of the working electrode, arising with the increase in HA. Above 3 mg, the presence of the huge amount of HA in the film reduced the conductivity of the electrode, as the inorganic material behaves like a physical barrier. The mass of 3 mg was therefore adopted for further experiments.

#### 3.2.4. Effect of pH on the Peak Current and Potential

**Figure 5:**
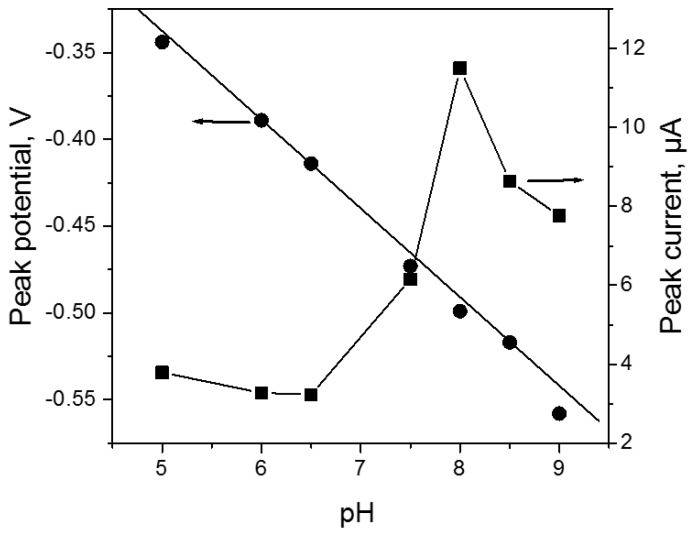
Effect of PBS solution pH on the peak current and peak potential of 10^−6^ M NBA at GCE/Lys/HA.

To elucidate the oxidation mechanism of NBA at the GCE/Lys/HA, the variation of peak potential with the pH was studied. For this purpose, DPV experiments at different pH values were carried out in the PBS, and the graphs of the peak current and peak potential vs. pH were plotted (Figure 5). As noticed, the peak current decreased slightly when the pH was raised from 5 to 6.5. Then, it significantly increased to reach a maximum value at pH 8; it then decreased for pH values set between 8 and 9. Regarding the plot of the peak potential vs. pH, a linear decreasing dependent relation was obtained, according to the equation Ep(V) = −0.074 − 0.053 pH (R^2^ = 0.995). The slope of −0.053 V/pH, obtained close to the theoretical value of −0.059 V/pH, showed that an equal number of protons and electrons were exchanged during this process [61,62,63].

**Scheme 2:**
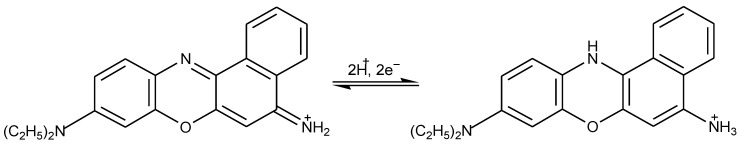
Mechanism of the electro-oxidation of NBA.

According to the structure of NBA and to the number of electrons and protons transferred, the following redox mechanism was proposed [59]. The chemical equation representing the electro-oxidation mechanism of NBA is highlighted in Scheme 2.

#### 3.2.5. Validation and Analytical Application of Lys/HA-Coated GCE Electrode Sensor—Calibration Curve and Interference Studies

**Figure 6:**
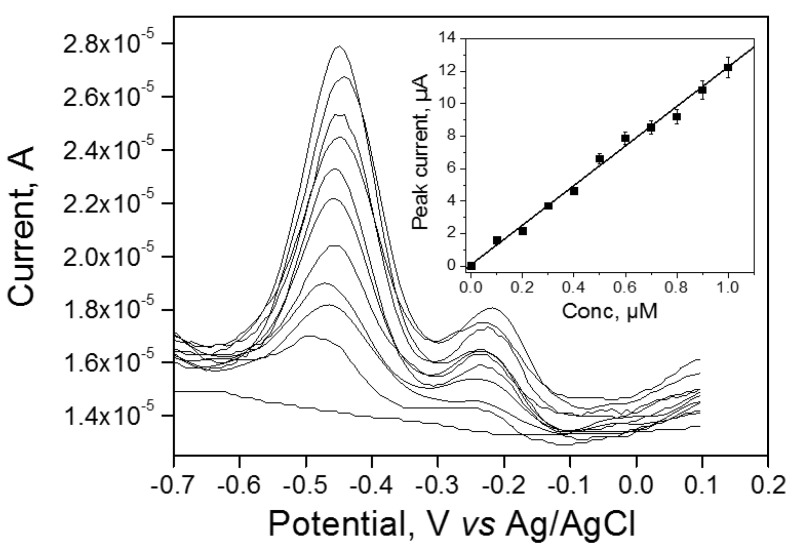
Differential pulse voltammetry (DPV) curves obtained on GCE/Lys/HA in PBS (pH 8) at various concentrations (0, 0.1, 0.2, 0.3, 0.4, 0.5, 0.6, 0.7, 0.8, 0.9, and 1 µM (DPV curves are ordered from bottom to top, respectively)) of NBA. The first peak, which is the most intense, was used to plot the concentration dependency. (The inset shows the corresponding calibration curve).

DPV analysis of NBA at various concentrations between 0.1 and 1 µM was performed using the GCE/Lys/HA under optimized conditions. The result is presented in Figure 6. As expected, the DPV peak current Ipa increased with NBA concentration (inset in Figure 6). The calibration equation and its correlation coefficient were Ipa(A) = 12.18022 [NBA] (mol L^−1^) + 5.3736 × 10^−8^ and R^2^ = 0.992, respectively. A limit of detection of 5.07 × 10^−8^ mol L^−1^ was determined, calculated as three times the standard deviation of the intercept divided by the slope of the calibration curve [64].

Table 3 gives a comparison with the values obtained in other articles for the detection of similar analytes, in particular methylene blue (MB), since to our knowledge, the electroanalysis of Nile blue A had not yet been carried out. The results displayed in this table highlight the fact that the developed sensor in this work highlighted a detection limit value comparable to those recorded in previous publications using modified electrodes for the detection of analytes similar to Nile blue A, in particular dyes.

The detection limit recorded was lower than those obtained by other authors for similar analytes. This showed that the Lys/HA composite made from less expensive, locally available and abundant materials is part of the efficient and stable electrode matrices allowing the simple and rapid modification of the electrode surface in order to improve its sensitivity and selectivity with respect to the electrochemical detection of dyes.

The selectivity of the GCE/Lys/HA was evaluated in the presence of some interfering molecules, such as toluidine blue (TB), methyl orange (MO), caffeine (CAF), citric acid (CA), and ascorbic acid (AA), as well as some metal cations (Ni^2+^, Pb^2+^, Cu^2+^, and Cd^2+^). Among its multiple applications, Nile blue A is a stain generally used in biology and histology. As examples, Nile blue A is mainly used with living cells, which fix it and give a blue color to cell nuclei [1]. It has also been applied to textiles and other products, such as leather, cosmetics, pulp and paper, pharmaceuticals, plastics, and foods [2,3,4,5]. In view of what was said previously, Nile blue A is very often used for various purposes and is therefore likely to be found in addition to other classes of chemical compounds in various types of liquid effluents, for example, biological, pharmaceutical, agro-food, and textile effluents. Concerning the study of the effect of interferents on the response of the Lys/HA fabricated electrode to the detection of Nile blue A, we attempted to reconstitute a physiobiological or industrial effluent by including a wide range of chemicals likely to be found in the effluents with Nile blue A at the same time. For this, we used as interference species other dyes (such as toluidine blue and methyl orange), compounds of pharmaceutical interest and also present in some foods consumed daily (caffeine, citric acid, and ascorbic acid), as well as metal ions (nickel, cadmium, copper, and lead ions). Thus, in the electrochemical cell containing a 10^−6^ M NBA solution, the previously mentioned species were added in a concentration equivalent to 0.5, 1, 5, and 10-fold that of NBA. The peak current of NBA was then recorded in optimized conditions, and its variation was noticed as shown in Table 4.

Globally, when added in a concentration 0.5-fold of NBA, all species did not interfere on the signal of the NBA analyte. At a concentration equivalent to that of NBA, the TB and AA significantly affected the response of NBA, as a variation in the target analyte signal for more than ±5% was recorded. Finally, when the concentration of the investigated species was more than five-fold that of NBA, then CAF, TB, and AA induced more deviation (≥7%) in the response of NBA, somewhat limiting the selectivity of the proposed sensor. For all the cationic species studied, the presence of these species more or less influenced the electrochemical signal of Nile blue A, when their concentration was 10 times higher than that of NBA in the reaction medium, with a recorded variation of more or less 5 % of the NBA target analyte signal. The obtained results suggest that a GCE/Lys/HA sensor could be efficiently applied for media less concentrated in NBA dye.

The practical application of the proposed sensor was evaluated through the analysis of NBA in a spring water sample collected in Dschang (Cameroon). The water was filtered using a filter paper, and then 50 mL was added in the electrochemical cell. The corresponding amounts of Na_2_HPO_4_ and KH_2_PO_4_ salts were added to reach a concentration of 0.1 M. A preliminary electrochemical experiment was performed to check the presence of NBA in spring water by using the procedure applied for calibration curve experiments. Under established optimized conditions, no NBA peak was found. By spiking the sample with 1 μM of NBA, recovery rates above 95% were achieved for experiments conducted in triplicate. This implies that the methodology herein proposed can be successfully used in the electroanalytical determination of NBA in water and other aqueous media expected to contain such a compound.

## 4. Conclusions

In this work, a composite material consisting of hydroxyapatite and L-lysine (Lys/HA) was prepared and tested as electrode material for the electroanalysis of Nile blue A (NBA). Before proposing the use of this composite for sensing purposes, its physicochemical properties were determined. The deposition of a thin coating of Lys/HA material on a glassy carbon electrode led to a sensitive and selective method for the detection of NBA based on differential pulse voltammetry (DPV). The proposed sensor electrode showed practical application in the detection of NBA in spring water.

## Figures and Tables

**Table 1:** Crystallite size, lattice strain, and crystallinity index of HA and Lys/Ha from W-H method.

Sample	Crystallite Size (nm)	Lattice Strain (10^−3^)	Crystallinity Index (%)
HA	21.68	3.2	42.4
Lys/HA	40.81	1.6	42.01

**Table 2:** Specific surfaces and pore volume of studied materials.

Sample	Surface Area (m^2^·g^−1^)	Pore Volume (cm^3^·g^−1^)
HA	46.69	0.1266
L-Lysine	0.23	-
Lys/HA	9.63	0.0258

**Table 3:** Comparison of the performances of the developed sensor GCE/Lys/HA with respect to the electroanalysis of Nile blue A, with those of other modified electrodes applied to the electroanalysis of similar analytes.

Electrode	Modifier	DLR (µM)	LOD (µM)	Method	Analyte	Reference
CPE ^(a)^	Thiol-functionalized clay	1–14	0.4000	CV ^(b)^	MB ^(c)^	[65]
CPE	Ibuprofen-coated gold	0.01–1	0.0039	DPV ^(d)^	MB	[66]
CPE	Coffee husks	1–125	3.0000	SWV ^(e)^	MB	[67]
GCE	CMTN ^(f)^	0.01–10	0.0030	DPV	MB	[68]
GCE ^(g)^	Lys/HA	0.1–1	0.0507	DPV	NBA	This work

^(a)^ Carbon paste electrode, CPE; ^(b)^ cyclic voltammetry, CV; ^(c)^ methylene blue, MB; ^(d)^ differential pulse voltammetry, DPV; ^(e)^ square wave voltammetry, SWV; ^(f)^ carbon-modified titanium dioxide nanostructured, CMTN; ^(g)^ glassy carbon electrode, GCE.

**Table 4:** Effect of some potential interference species on the response of the GCE/Lys/HA to 10^−6^ mol·L^−1^ NBA in 0.1 M PBS (at pH 8).

Interference Species	Added Amount over NBA Concentration	Percentual Variation in the Anodic Peak Current (I_pa_) for NBA
Toludine blue	0.5	−3.45
	1	−24.48
	5	−44.15
	10	−68.49
Methyl orange	0.5	−1.68
	1	0
	5	−7.95
	10	−3.9
Caffeine	0.5	0.72
	1	−4.08
	5	−7.59
	10	−55.75
Citric acid	0.5	−6.34
	1	−2.21
	5	0
	10	18.03
Ascorbic acid	0.5	−1.45
	1	8.13
	5	13.44
	10	18.78
Pb^2+^	0.5	1.69
	1	1.37
	5	−2.23
	10	−8.8
Cu^2+^	0.5	1.12
	1	2.34
	5	−4.3
	10	−6.48
Ni^2+^	0.5	0.32
	1	1.31
	5	4.09
	10	5.16
Cd^2+^	0.5	−0.28
	1	−2.86
	5	−3.67
	10	−7.5

## Data Availability

Not applicable.

## Supplementary Materials

The following supporting information can be downloaded at: https://www.mdpi.com/article/10.3390/ma15124262/s1, Figure S1: Williamson–Hall plots for (a) Lys/HA and (b) HA, Figure S2: Plots of the anodic and cathodic peak currents as a function of the scan rate, recorded on GCE/Lys/HA in 0.1 M PBS (pH = 5.5) containing 1 mM of NBA. The scan rate was varied between 15 and 150 mV.s^−1^. Figure S3: Effect of HA mass in the Lys/HA film on the DPV peak current of 1µM of NBA in 0.1 M PBS (pH 5.5).
