# Supplementary material for: Hydroxyapatite/L-Lysine Composite Coating as Glassy Carbon Electrode Modifier for the Analysis and Detection of Nile Blue A
Source: Materials (Basel). 2022 Jun 16;15(12):4262. doi: 10.3390/ma15124262 (PMC9230729; doi:10.3390/ma15124262)
Supplement: Supplementary file 1 [file materials-15-04262-s001.zip › materials-1748302-supplementary.pdf]

SUPPLEMENTARY MATERIAL

Hydroxyapatite/L-Lysine Composite Coating as Glassy Carbon Electrode Modifier for the Analysis and Detection of Nile Blue A

Jimmy Julio Kouanang Ngouoko [1], Kevin Yemele Tajeu [1], Ranil Clément Tonleu Temgoua [1,2], Giscard Doungmo [3], Ingo Doench [4,5,6], Arnaud Kamdem Tamo [4,5,6,*], Théophile Kamgaing [1], Anayancy Osorio-Madrazo [4,5,6,*] and Ignas Kenfack Tonle [1,*]

[1] Department of Chemistry, Faculty of Science, University of Dschang, Dschang P.O. Box 67, Cameroon; ngouoalex@yahoo.com (J.J.K.N.); tasergekev@yahoo.fr (K.Y.T.); raniltemgoua@yahoo.fr (R.C.T.T.); theokamgaing@yahoo.fr (T.K.)

[2] Higher Teacher Training College, University of Yaoundé 1, Yaoundé P.O. Box 47, Cameroon

[3] Institute of Inorganic Chemistry, Christian-Albrechts-Universität zu Kiel, Max-Eyth-Straβe 2, 24118 Kiel, Germany; gdoungmo@ac.uni-kiel.de (G.D.)

[4] Laboratory for Bioinspired Materials BMBT, Institute of Microsystems Engineering IMTEK-Sensors, University of Freiburg, 79110 Freiburg, Germany; ingo.doench@imtek.uni-freiburg.de (I.D.)

[5] Freiburg Center for Interactive Materials and Bioinspired Technologies FIT, University of Freiburg, 79110 Freiburg, Germany

[6] Freiburg Materials Research Center FMF, University of Freiburg, 79104 Freiburg, Germany

[*] Correspondence: arnaud.kamdem@imtek.uni-freiburg.de (A.K.T.); anayancy.osorio@imtek.uni-freiburg.de (A.O.-M.); ignas.tonle@univ-dschang.org (I.K.T.); Tel.: +49-761-203-95096 (A.K.T.); +49-761-203-67363 (A.O.-M.); +237-696-141-545 (I.K.T.)

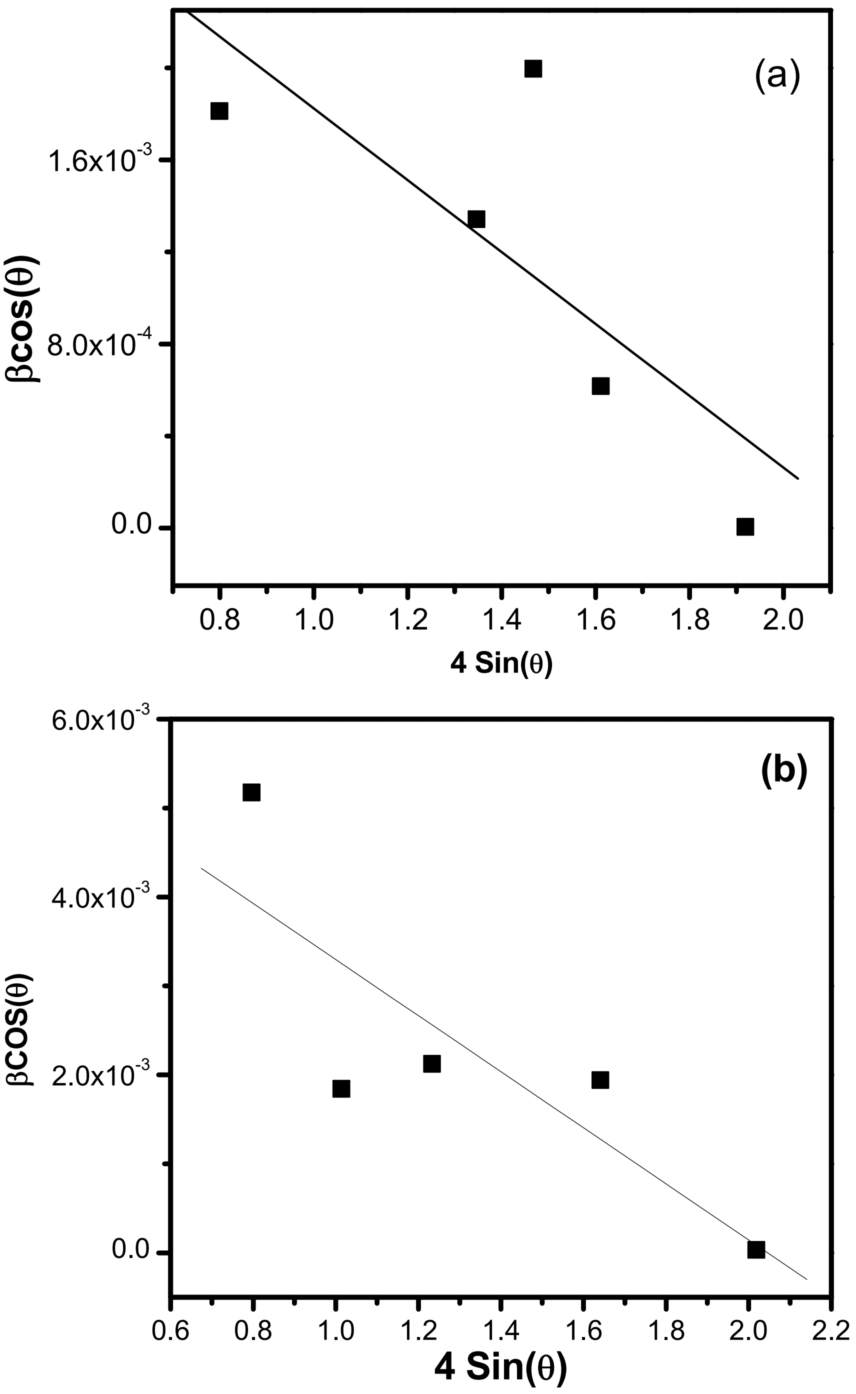

Figure S1. Williamson-Hall plots for (a) Lys/HA and (b) HA.

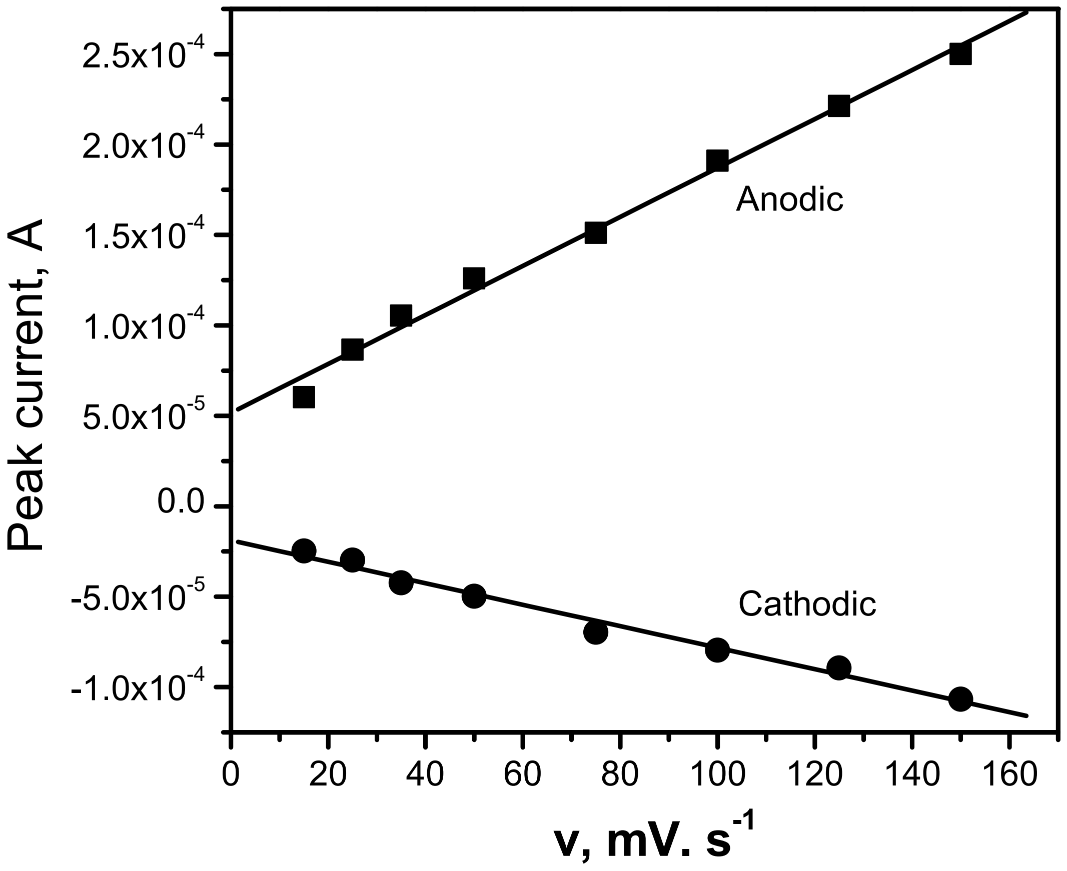

Figure S2. Plots of the anodic and cathodic peak currents as a function of the scan rate, recorded on GCE/Lys/HA in 0.1 M PBS (pH = 5.5) containing 1 mM of NBA. The scan rate was varied between 15 and 150 $mV.s^{-1}$.

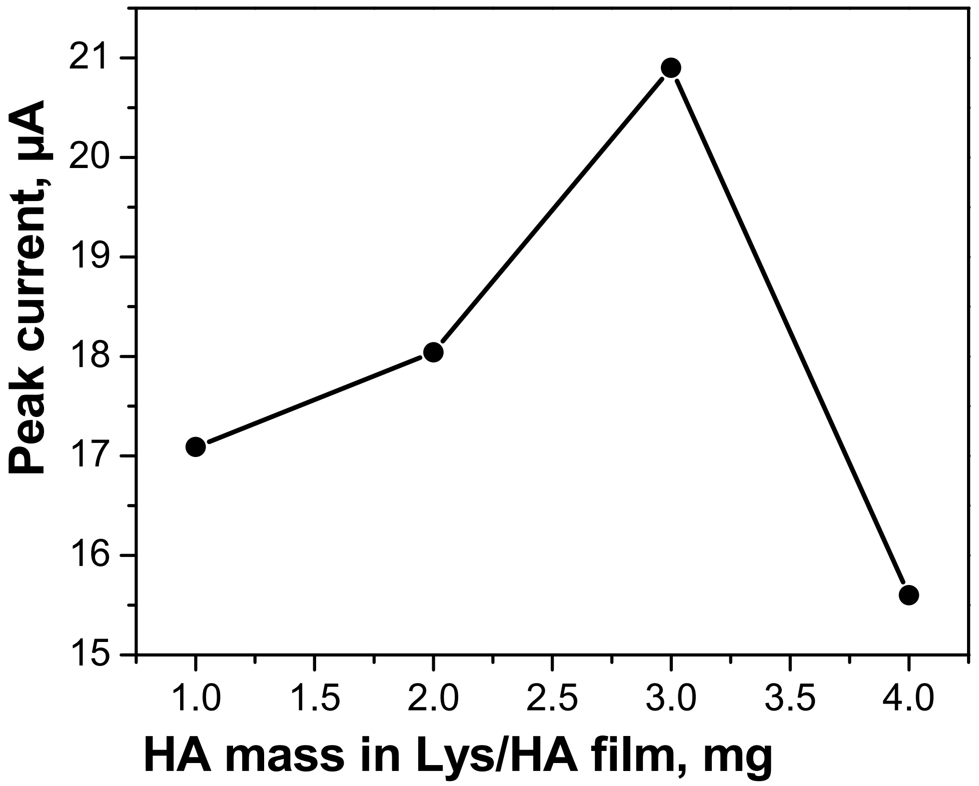

Figure S3. Effect of HA mass in the Lys/HA film on the DPV peak current of 1µM of NBA, in in 0.1 M PBS (pH 5.5).
